# Identifying Rural Hotspots for Head and Neck Cancer Using the Bayesian Mapping Approach

**DOI:** 10.3390/cancers17050819

**Published:** 2025-02-26

**Authors:** Poornima Ramamurthy, John Adeoye, Siu-Wai Choi, Peter Thomson, Dileep Sharma

**Affiliations:** 1 College of Medicine and Dentistry, James Cook University, Smithfield, QLD 4878, Australia; poornima.ramamurthy@jcu.edu.au (P.R.);; 2 Oral and Maxillofacial Surgery, Faculty of Dentistry, The University of Hong Kong, Hong Kong, China; 3 School of Health Sciences, The University of Newcastle, Ourimbah, NSW 2258, Australia

**Keywords:** rural and remote, cancer, epidemiology, Bayesian mapping

## Abstract

**Background**: The Bayesian mapping approach has not been used to identify head and neck cancer hotspots in Australia previously. This study aims to identify rural communities at risk of head and neck cancer (HNC) for targeted prevention programs. **Methods**: This study included data from 23,853 cases recorded in the Queensland Cancer Register between 1982 and 2018. Outcomes for mapping included incidence, overall mortality, 3-year mortality, and 5-year mortality. Local government areas (LGAs) with a general population aged 15 years and above (according to 2016 census data from the Australian Bureau of Statistics) were utilized for mapping. **Results**: Of the 59 LGAs with higher-than-average risk, 22 predominantly rural and remote LGAs showed statistically significant higher risks of head and neck cancer occurrence. Estimated median standardized mortality ratios (SMRs) ranged from 0.57 to 3.44 and were higher than the state average in 38 LGAs. Four LGAs had the highest mortality—the Shires of Quilpie, Yarrabah, Murweh, and Hinchinbrook. **Conclusions**: Whilst reasons for some LGAs exhibiting higher HNC are unknown, Bayesian mapping highlights these rural and remote regions as worthy of further investigation. In conclusion, the Bayesian disease mapping approach is effective in identifying high-risk communities for HNC. Findings from this study will aid in designing targeted screening and interventions for the prevention and management of head and neck cancer in regional and remote communities through support services such as a cancer navigator.

## Simple Summary

Cancers often tend to occur at a higher rate in rural and remote communities due to various reasons. Identifying these cancer hotspots will assist in adequate resourcing of such hotspots. This study was conducted to identify head and neck cancer hotspots in Queensland (QLD), Australia, based on the historical data collected by the cancer register and employing a specialized Bayesian mapping approach. The findings of this study suggested that many rural and remote regions in QLD experience significantly higher head and neck cancer incidence rates and death rates when compared to the QLD state average rates and their surrounding regions. Additionally, a generalized increasing trend of head and neck cancers was noted across the studied period (1982–2018). Although the precise reasons for this increasing trend over time are unclear, a range of factors, such as distance from the tertiary hospitals, lack of awareness of risk factors, behavioral and lifestyle factors, and delayed diagnosis, may be considered to contribute. Our study successfully utilized a robust method to identify head and neck cancer hotspots and will aid in supporting the community and healthcare providers in the region with additional resources to prevent and manage cancer.

## 1. Introduction

Cancers in the head and neck (HNC) region are known to originate from the mucosal lining of the oral cavity, oropharynx, hypopharynx, larynx, nasopharynx, and sinonasal tract [1]. HNCs are the seventh most common type of cancer in the world, with 660,000 new cases accounting for 5% of total global cancer cases [2,3].

In Australia, 95% of head and neck cancers are squamous cell carcinomas (SCCs) involving the mucosa of the oral cavity, pharynx, and larynx and account for 2–3% of all cancers annually [4,5]. The highest rates of HNC are reported among people living in the Northern Territory with a significant proportion of communities being Aboriginal people and living in rural and remote areas of the state [6]. HNC is known to be a lethal form of cancer as over 50% of diagnosed cases are often diagnosed in the advanced stages of the disease [7]. It is more common in males than females in Australia, and oropharyngeal cancer accounts for nearly 3% of all cancer types [7,8]. Generally, the survival rates are higher due to advancements in treatment approaches and better access in urban areas, but there is a significant reduction in the survival rates of the rural population due to limited access to tertiary care in rural and remote regions [7,9,10]. Additionally, head and neck cancer 10-year observed survival rate for Indigenous Australians (26.5%) is significantly lower than that for non-Indigenous Australians (47.4%), which is a concerning finding [11]. Particularly, Indigenous people living in regional areas are more likely to be diagnosed at an advanced stage of cancer and less likely to receive optimal treatment due to a lack of awareness about the severity of the disease and limited access to healthcare facilities [10,12,13]. This puts the already disadvantaged population at high risk, warranting further screening, education, and awareness programs to facilitate early detection and treatment, thereby improving the overall quality of life.

Bayesian disease mapping is a specialized epidemiological approach used to identify and quantify variations and patterns of diseases among small population areas in a country or state [14,15]. Compared to traditional methods of estimating incidence and mortality ratios in small areas that can provide a value of zero or be susceptible to misinterpretations in areas with small populations, the Bayesian model can provide reliable incidence/mortality estimates by combining observed data (likelihood function) and predicted uncertainty as a measure of one’s belief for the estimates before the data are analyzed (prior distribution). This approach has been an effective aid in identifying communities at higher risk of HNC, so that resources and targeted public health screening and healthcare interventions can be optimally planned [14]. Our research team used the Bayesian mapping approach on oral and oropharyngeal carcinomas in Australia recently [16]. However, this approach has not been used to identify HNC hotspots in any of Australia’s states with a dataset containing 36 years of cancer data. Hence, this retrospective study was conducted to map and identify HNC hotspots in Queensland, Australia, using the Bayesian mapping approach.

## 2. Material and Methods

### 2.1. Data Collection

Data held by the Queensland Cancer Register (QCR) was accessed for the period 1982–2018 after relevant approvals from the Human Research Ethics Committee at James Cook University (#H8609) and Queensland Health under the Public Health Act 2005. All the data were managed within the Australian Code for the Responsible Conduct of Research.

### 2.2. Data Analysis

Descriptive statistics were performed and presented as tables and text. Head and neck cancer was broadly classified based on its location into cancers involving the external lip, oral cavity, oropharynx, nasopharynx, hypopharynx, larynx, nasal cavity, paranasal sinuses, and salivary glands. This was conducted according to the International Classification of Diseases (ICD-10) codes C00–C14, C30.0, C31, and C32. Outcomes for mapping included the incidence, overall mortality, 3-year mortality, and 5-year mortality of head and neck cancers in Queensland, Australia. Queensland suburbs and localities (SSC) were the spatial units in the original cohort dataset extracted. These SSCs were used to generate higher areal data at the level of the local government areas (LGAs, *n* = 78) from the 2016 Australian census.

To calculate the expected incidence and mortality of head and neck cancer, this study used the LGA population of individuals aged above 15 years based on the 2016 census data recorded by the Australian Bureau of Statistics [1]. The binary adjacency matrix for determining neighborhood dependence and common land boundaries was created using GeoDa (v1.20) with the rook definition. One LGA (Mornington Shire) did not have neighbors during adjacency matrix creation since it comprises islands with no common land boundary. In line with previous adjustments performed for islands in Australia by Duncan et al. [17], the closest neighbor, i.e., Burke Shire, was manually assigned by the team before spatial analysis.

The spatial modeling approach, parameter selection, hyperprior selection, and sensitivity analysis were inspired by our previous analysis of oral cancer data in Hong Kong and Queensland [14,16,18]. In detail, the fully Bayesian method was used to estimate the adjusted incidence and mortality of head and neck cancer, which was implemented using the Gibbs sampling type of Markov Chain Monte Carlo (MCMC) algorithm to sample parameter estimates. Also, a single Markov chain was run for incidence and mortality models. The Besag, York, and Mollie (BYM) method was used to model the incidence and mortality of head and neck cancer. The overall risk effect was assigned vague normal distributions with a mean of 0 and a variance of 10^6^, and conditional autoregressive (CAR) priors were specified to model spatial effects. Noninformative gamma-distributed hyperpriors were also specified in the CAR priors and unstructured effects of the BYM model. The rationale for selecting the BYM method was according to our previous spatial analysis for oral cancer [18], where the model had a lower deviance information criterion and Watanabe–Akaike information criterion compared to the Leroux model.

Overall, 500,000 iterations were conducted with a thinning interval of 20 to ensure independent estimates were sampled. For the incidence and mortality models, the chain had a burn-in of 300,000 iterations to ensure that all nodes converged. The 200,000 iterations that followed were then taken, leading to a sample size of 10,000 estimates. The convergence of the Markov chain was assessed by visualizing the trace, density, and autocorrelation plots and using Geweke’s convergence diagnostics.

Median estimates of smoothed incidence and mortality risks were used to draw choropleth maps using the open-source geometry data and shapefiles obtained from the Queensland Government Open Data Portal [19]. LGA boundaries information was also provided by the Queensland Government Department of State Development, Infrastructure, Local Government, and Planning (https://www.statedevelopment.qld.gov.au/__data/assets/pdf_file/0019/42454/local-government-area-boundaries.pdf, accessed on 20 July 2024). Spatial clustering or variation was assessed using the global and local indicator of spatial autocorrelation (LISA)/local Moran’s I test, as its sensitivity for quantifying autocorrelation in cancer incidence/mortality estimates have been shown in previous studies [14,16,18,20]. The probability values of these tests were then estimated using Monte Carlo randomization with 99,999 permutations. When defining spatial clusters or outliers, the lower limit of the 95% credible interval of the smoothed median relative risks was used to determine areas with statistically significant high or low risk for oral cancer incidence and mortality. Statistical and spatial analyses as well as cartography were performed in SPSS v27, R statistical software, WinBUGS, and GeoDa v1.20.

## 3. Results

### 3.1. Patient Cohort

**Figure 1:**
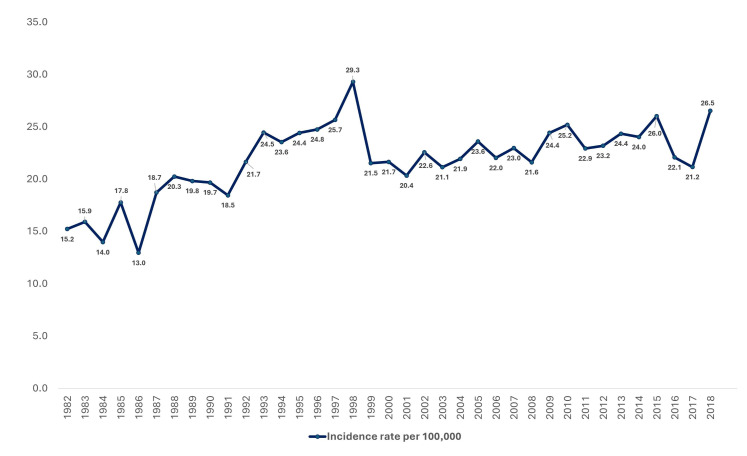
Longitudinal trend of head and neck cancer incidence in Queensland from 1982 to 2018.

**Figure 2:**
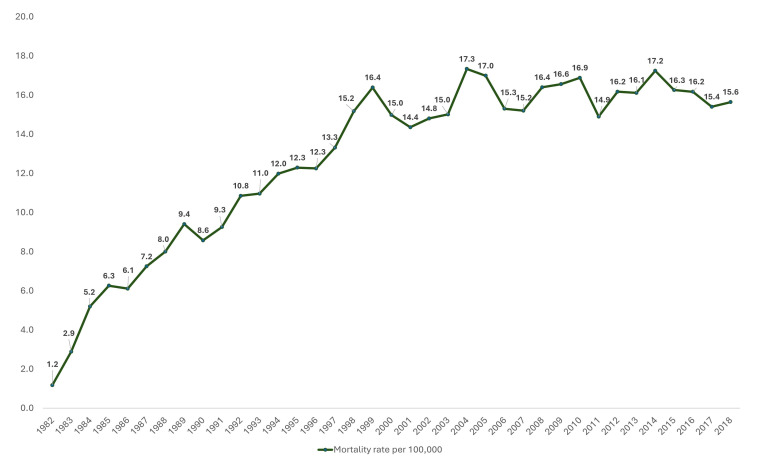
Longitudinal trend of head and neck cancer mortality in Queensland from 1982 to 2018.

In total, 23,917 patients with head and neck cancers were diagnosed and treated between 1982 and 2018 in Queensland, Australia. Of these, sixty-four patients were excluded from further analysis due to the following reasons: patients aged below 15 years (n = 5), patients with branchial cleft tumors (n = 2), and patients with tumors involving unspecified head and neck sites (n = 57). Consequently, 23,853 patients with HNC were included in the spatial analysis. The age of included patients ranged from 15 to 105 years with a median age (IQR) of 62 (53–71) years. Most patients were between 40 and 64 years old (50.6%), and more males than females (77.5% vs. 22.5%) were noted in the cohort (Table 1). Regarding the site of occurrence, most participants presented with lip cancer (27.3%), oral cavity cancer (25.8%), oropharyngeal cancer (21.5%), and laryngeal cancer (16.9%). As of 1 May 2022, only 40.8% of the patients were alive, and patients’ survival times ranged from <1 month to 483 months with a median survival time (IQR) of 81 (27–162) months. Furthermore, 8338 patients (35%) died within five years, while 6714 (28.2%) patients died within three years of their diagnosis. Longitudinal trends of head and neck cancer incidence and mortality rates from 1982 to 2018 are shown in Figure 1 and Figure 2. Head and neck cancer incidence was the highest in 2008, with 29.3 new cases per 100,000, while head and neck cancer mortality was the highest in 2004 (17.3 per 100,000) and 2014 (17.2 per 100,000).

### 3.2. Incidence Risk Mapping

**Figure 3:**
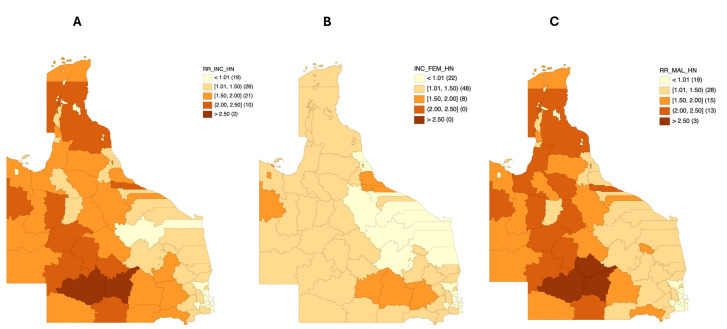
Bayesian smoothed median SIR maps for (**A**) head and neck cancer incidence relative risks in all 78 Queensland LGAs, (**B**) head and neck cancer incidence relative risks among females in all 78 Queensland LGAs, and (**C**) head and neck cancer incidence relative risks among males in all 78 Queensland LGAs.

HNC incidence mapping included 23,853 patients residing in Queensland. The median smoothed standardized incidence ratio (SIR) estimated relative to the population differences in the LGAs ranged from 0.48 in Palm Island Aboriginal Shire to 2.74 in Quilpie Shire (Figure 3A). In comparison to the Queensland average median SIR (1.00), 59 LGAs had a higher risk; however, considering the lower limit of the 95% credible interval (CI), 22 LGAs had significantly higher risks of head and neck cancer occurrence (supplementary data). Twelve LGAs had higher incidence risks exceeding 100% of the Queensland average, while 30 LGAs had incidence risks that were between 1% and 100% higher than the state average. Only Murweh Shire had median smoothed SIR estimates and a 95% CI that was consistently higher than 100% of the Queensland average (2.61 [2.03–3.28]).

On stratifying head and neck cancer by gender, the median smoothed SIRs for females and males ranged from 0.73 to 1.87 and from 0.54 to 2.80, respectively (Figure 3B,C). For females, 11 LGAs had a significant incidence risk, all of which had median smoothed SIRs between 1% and 100% higher than the average risk (1.16–1.87). The highest incidence risks were observed in Mount Isa City (1.87 [1.38–2.49]). For males, 42 LGAs had significantly higher excess incidence risks than the state average, with a risk distribution similar to that of the total head and neck cancer patients (supplementary data). Twenty-six LGAs had median smoothed SIRs that were between 1% and 100% higher than the state average, while 16 LGAs had a median SIR > 100% of the state average. Likewise, only Murweh Shire had a median smoothed SIR and a 95% CI that was consistently above 100% of the state average (2.69 [2.05–3.49]).

### 3.3. Incidence Risk Mapping by Different Anatomical Sites

**Figure 4:**
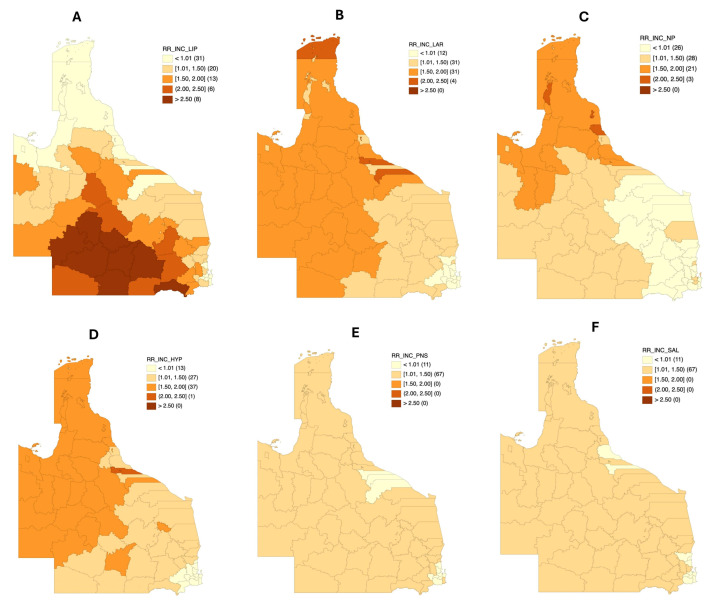
Bayesian smoothed median SIR maps for (**A**) lip cancer incidence risks in 78 Queensland LGAs, (**B**) laryngeal cancer incidence risks in 78 Queensland LGAs, (**C**) nasopharyngeal cancer incidence risks in 78 Queensland LGAs, (**D**) hypopharyngeal cancer incidence risks in 78 Queensland LGAs, (**E**) incidence risks for nasal cavity and paranasal sinus malignancies in 78 Queensland LGAs, and (**F**) salivary gland cancer incidence risks in 78 Queensland LGAs.

Different patterns in the incidence risk distributions among various LGAs were observed based on the anatomical sites affected by cancer (Figure 4A–F). For lip cancers, median smoothed SIRs ranged from 0.34 to 3.41. Twenty-seven LGAs had a significantly higher incidence risk of lip cancer compared to the Queensland average. Of these LGAs, 13 had excess relative risks between 1% and 100% higher than the state average (1.27–1.77), while 14 had relative incidence risks exceeding 100% of the state average (2.12–3.41). Only Murweh Shire (3.41 [2.33–4.89]) and Maranoa Regional Council (2.88 [2.22–3.71]) had incidence risks that were constantly higher than 100% of the state average according to the lower limit of the 95% credible interval.

Regarding laryngeal cancers, the median smoothed SIR ranged between 0.65 and 2.23 (Figure 4B). Likewise, 33 LGAs had a significantly higher incidence risk for this cancer relative to their total populations and the Queensland average. Only 4 of 33 LGAs had median smoothed SIRs that were above 100% of the state average (2.02–2.23). These LGAs in ascending order of incidence risk distribution were Burdekin Shire, Hinchinbrook Shire, Torres Shire, and Yarrabah Aboriginal Shire. Likewise, 29 LGAs had higher incidence risks ranging between 1% and 100% (1.21–1.95). No LGA had consistent incidence risk estimates exceeding 100% of the state average.

The distribution of median smoothed estimates for nasopharyngeal cancer incidence in Queensland is shown in Figure 4C. Incidence estimates for this cancer type ranged from 0.62 to 2.50. Only three LGAs (Mareeba Shire, Hinchinbrook Shire, Douglas Shire) had a significantly higher risk of nasopharyngeal cancer incidence above the state average. The median smoothed SIR of these LGAs was from 1.84 to 2.02, and none of them consistently had higher excess relative risks above 100% of the state average (supplementary data).

Incidence risks for hypopharyngeal cancers (Figure 4D) were found to be between 0.69 and 2.04. Nineteen LGAs had significantly higher median smoothed SIR estimates for this cancer type. Seventeen out of 19 LGAs had median incidence estimates between 1% and 100% higher than the state average risk (1.41–1.93), while two LGAs (Hinchinbrook Shire and Northern Peninsula Area Regional Council) had median incidence estimates above 100% higher than the state average risk (2.00–2.04). None of the significant LGAs had credible intervals with the same risk distribution as the median incidence risk estimates.

The median smoothed SIR for nasal cavity and paranasal sinus cancers, as well as salivary gland cancers, was from 0.87 to 1.28 and 0.75 to 1.28, respectively (Figure 4E,F). None of the LGAs had significant incidence risks of these malignancies that were consistently higher than the Queensland average. Thus, these malignancies have low or equivocal risks in the different LGAs of Queensland.

### 3.4. Autocorrelation Analysis for Head and Neck Cancer Incidence

**Figure 5:**
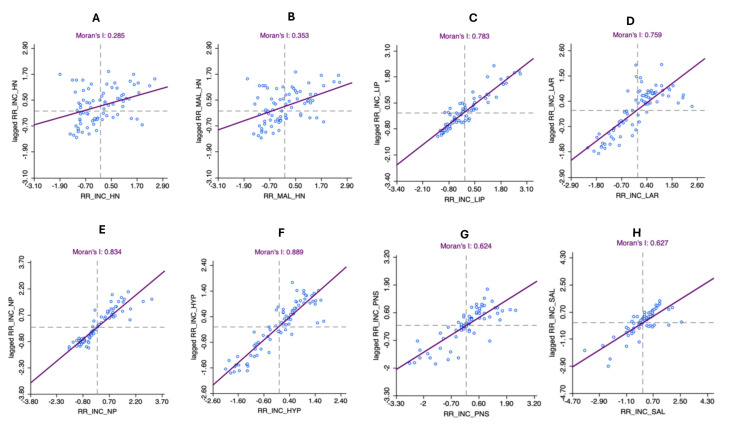
Scatter plots for global Moran’s I estimation for (**A**) median smoothed SIRs of head and neck cancer in 78 Queensland LGAs, (**B**) median smoothed SIRs of head and neck cancer among males in 78 Queensland LGAs, (**C**) median smoothed SIRs of lip cancer in 78 Queensland LGAs, (**D**) median smoothed SIRs of laryngeal cancer in 78 Queensland LGAs, (**E**) median smoothed SIRs of nasopharyngeal cancer in 78 Queensland LGAs, (**F**) median smoothed SIRs of hypopharyngeal cancer in 78 Queensland LGAs, (**G**) median smoothed SIRs of nasal cavity and paranasal sinus cancers in 78 Queensland LGAs, and (**H**) median smoothed SIRs of salivary gland cancer in 78 Queensland LGAs. Dotted lines in the scatter plots represent the x and y axes of the cartesian plane.

Global Moran’s I (GMI) scatter plots for the incidence risk estimates are shown in Figure 5A–H. The overall incidence risk displayed a significant positive spatial autocorrelation of 0.285 (*p* < 0.001), which was also observed among males (GMI statistic = 0.353; *p* < 0.001). The different cancer types also displayed significant spatial clustering with a GMI statistic that ranged from 0.624 for nasal cavity and paranasal sinus cancers to 0.889 for hypopharyngeal cancers (*p* < 0.001).

**Figure 6:**
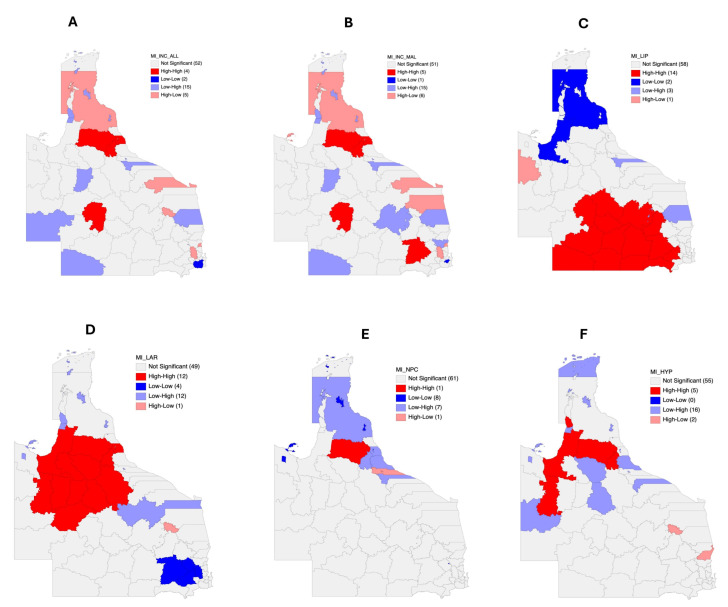
Local index of spatial autocorrelation (LISA) maps for (**A**) significant median smoothed SIRs of head and neck cancer in 78 Queensland LGAs, (**B**) significant median SIRs of head and neck cancer among males in 78 Queensland LGAs, (**C**) significant median smoothed SIRs of lip cancer in 78 Queensland LGAs, (**D**) significant median smoothed SIRs of laryngeal cancer in 78 Queensland LGAs, (**E**) significant median smoothed SIRs of nasopharyngeal cancer in 78 LGAs, and (**F**) significant median smoothed SIRs of hypopharyngeal cancer in 78 Queensland LGAs. The terminology “high-high” denotes areas of high incidence located adjacent to another high-incidence area. Similarly, “low-high” denotes areas of high incidence located adjacent to an area with low incidence, and so on.

Further analysis to identify spatial clusters and outliers found that higher head and neck cancer incidence risks were clustered around Longreach and Tablelands Regional Councils as well as Mareeba Shire (Figure 6A). In addition to the aforementioned LGAs, Western Downs and Cairns Regional Councils also formed high-risk spatial clusters with their neighbors for head and neck incidence among males (Figure 6B).

Fourteen LGAs that formed significant high-risk clusters for lip cancer were identified along with some of their neighbors (Figure 6C). These included Blackall Tambo, Central Highlands, Goondiwindi, Longreach, Maranoa, Toowoomba, and Western Downs Regional Councils, as well as Barcaldine, Banana, Balonne, Murweh, Paroo, Quilpie, and Bulloo Shires. Likewise, 12 significant high-risk LGA clusters were identified for laryngeal cancers, which included Etheridge Shire, Flinders Shire, Mareeba Shire, McKinlay Shire, Richmond Shire, Winton Shire, Carpentaria Shire, Cloncurry Shire, Croydon Shire, Cairns Regional Council, Charters Towers Regional Council, and Tablelands Regional Council, as well as some of their neighbors (Figure 6D).

Mareeba Shire was the only LGA that formed high-risk clusters with some of its neighbors regarding nasopharyngeal cancer incidence (Figure 6E). For hypopharyngeal cancer, five high-high spatial clusters were found which included Mareeba, Douglas, Cloncurry, and Carpentaria Shires, as well as Tablelands Regional Council (Figure 6F). Low-low spatial clusters were only identified for the incidence risks of nasal cavity and paranasal sinus malignancies as well as salivary gland cancers.

### 3.5. Mortality Risk Mapping

**Figure 7:**
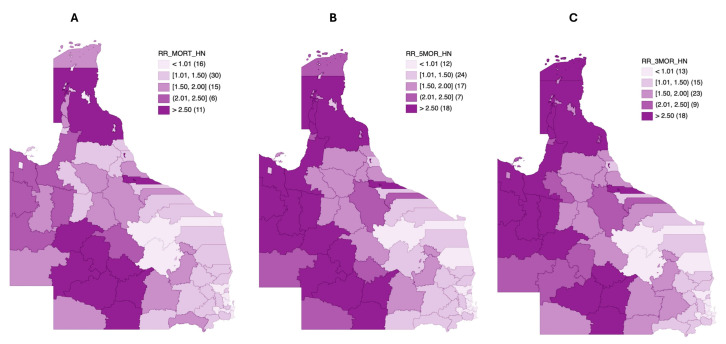
Bayesian smoothed median SMR maps for (**A**) head and neck cancer overall mortality risks in all 78 Queensland LGAs, (**B**) head and neck cancer five-year mortality risks in all 78 Queensland LGAs, and (**C**) head and neck cancer three-year mortality risks in all 78 Queensland LGAs.

Choropleth maps based on the median smoothed standardized mortality ratio (SMR) of head and neck cancer in Queensland are shown in Figure 7A–C. The median smoothed SMR for head and neck cancer overall mortality was from 0.57 to 3.44. Thirty-eight LGAs had significantly higher-than-state-average (SMR = 1.00) mortality risks. Furthermore, 21/38 LGAs had higher median smoothed SMRs, which were between 1% and 100% over the state average (1.09–1.91), while 17 LGAs had mortality estimates that were above 100% of the state average (2.03–3.44). Of these LGAs with the highest mortality estimates, Quilpie Shire (3.44 [2.07–5.58]), Yarrabah Aboriginal Shire (3.17 [2.05–4.67]), Murweh Shire (3.08 [2.28–4.07]), and Hinchinbrook Shire (2.54 [2.06–3.09]) consistently displayed credible intervals that were also >100% of the state average.

Regarding the five-year mortality of head and neck cancer in Queensland (Figure 7B), median smoothed SMRs ranged from 0.59 to 5.09 with 41 LGAs having significant mortality risks. Also, 23 of 41 LGAs had median smoothed SMRs that were > 100% higher than the state average (2.12–5.09; supplementary data). Considering the 95% CIs, the highest mortality risks five years after cancer diagnosis were observed in Yarrabah Aboriginal Shire (5.09 [3.23–7.58]), Cook Shire (3.39 [2.37–4.74]), Murweh Shire (3.07 [2.10–4.31]), Mount Isa City (2.77 [2.25–3.38]), and Hinchinbrook Shire (2.69 [2.08–3.44]).

Further stratification based on mortality three years after diagnosis produced median smoothed SMRs that ranged from 0.60 to 5.64. Of the 78 LGAs, 40 had a significantly higher risk of mortality from head and neck cancers 3 years following diagnosis. Also, 23 of the 40 LGAs with significant mortality risks had a risk level that was above 100% of the Queensland average (2.23–5.64). The highest 3-year mortality risks considering the credible intervals and population distribution were observed in seven LGAs. These included Yarrabah Aboriginal Shire (5.64 [3.50–8.63]), Cook Shire (3.44 [2.37–4.90]), Winton Shire (3.40 [2.00–5.76]), Carpentaria Shire (3.21 [2.00–5.08]), Murweh Shire (3.10 [2.07–4.52]), Mount Isa City (2.93 [2.33–3.62]), and Hinchinbrook Shire (2.69 [2.02–3.53]).

### 3.6. Autocorrelation Analysis for Head and Neck Cancer Mortality

**Figure 8:**
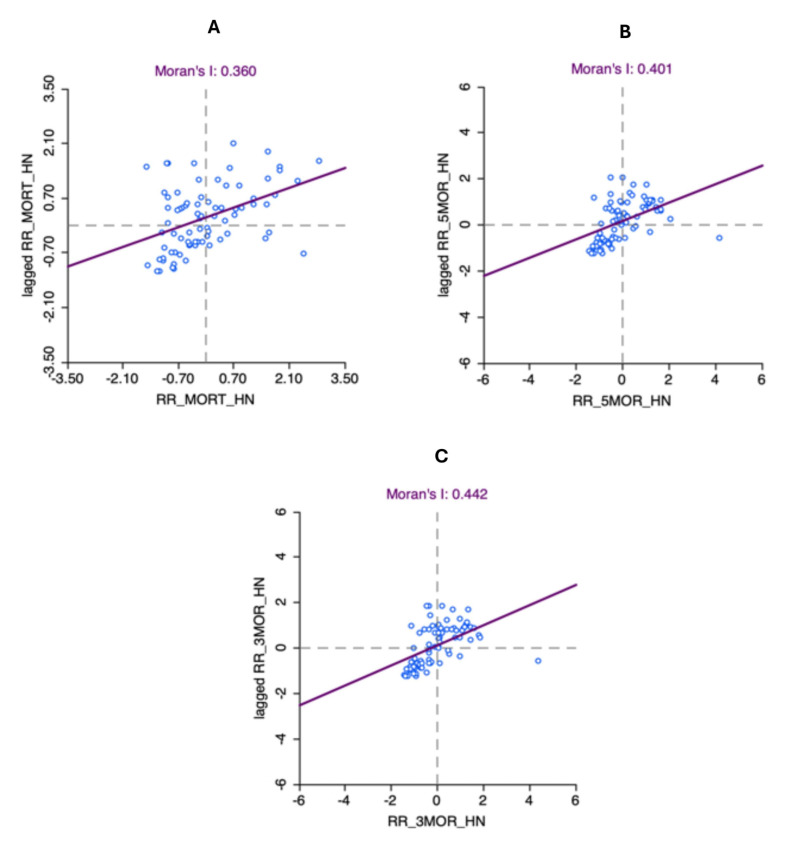
Scatter plots for global Moran’s I estimation for (**A**) median smoothed overall SMRs of head and neck cancer in 78 Queensland LGAs, (**B**) median smoothed five-year SMRs of head and neck cancer in 78 Queensland LGAs, and (**C**) median smoothed three-year SMRs of head and neck cancer in 78 Queensland LGAs. Dotted lines in scatter plots represent the x and y axes of the cartesian plane.

The three mortality measures (i.e., overall mortality, 5-year mortality, and 3-year mortality) each showed a significant positive spatial autocorrelation with GMI statistics that ranged from 0.360 to 0.442 (*p* < 0.001; Figure 8A–C).

**Figure 9:**
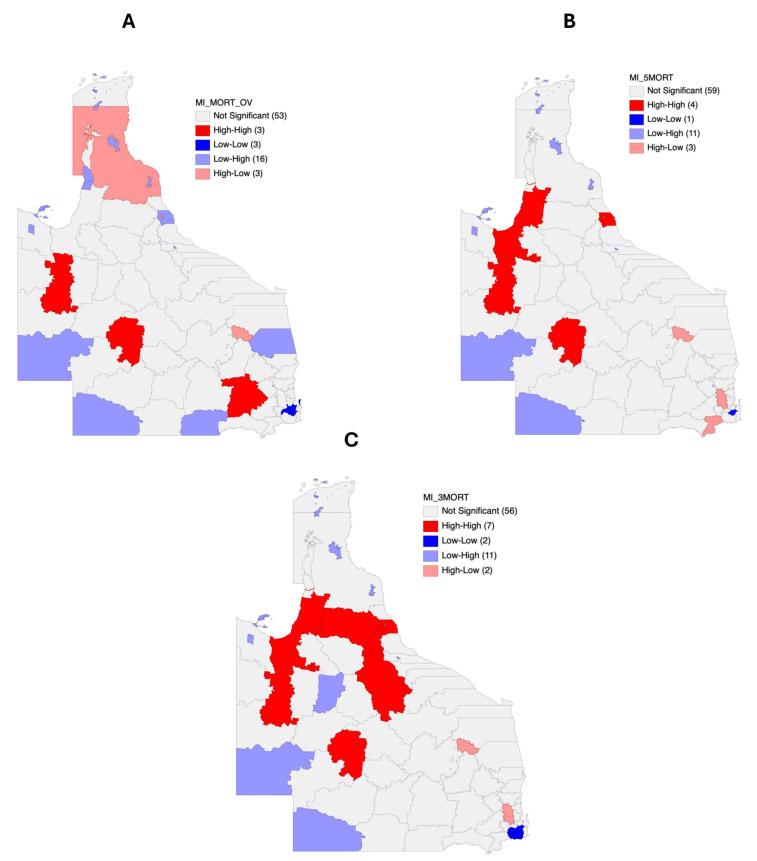
Local index of spatial autocorrelation (LISA) maps for (**A**) significant median overall SMRs of head and neck cancer in all 78 Queensland LGAs, (**B**) significant median five-year SMRs of head and neck cancer in all 78 Queensland LGAs, and (**C**) significant median smoothed three-year SMRs of head and neck cancer in all 78 Queensland LGAs.

Further, local index of spatial autocorrelation (LISA) maps for each of the mortality estimates are presented in Figure 9A–C. Three LGAs (Longreach, Western Downs, and Cloncurry Shire) and their neighbors formed high-risk clusters for overall head and neck cancer mortality. For five-year mortality, LISA analysis identified four significant high-risk LGA clusters around Cairns, Longreach, Carpentaria Shire, and Cloncurry Shire (Figure 9B). Furthermore, in addition to these four LGAs, three more LGAs, i.e., Mareeba Shire, Tablelands Regional Council, and Charters Towers Regional Council, formed significant high-risk mortality clusters with their neighbors regarding the three-year mortality of head and neck cancer (Figure 9C).

## 4. Discussion

Cancer is a serious and increasing health issue worldwide, including some forms of HNC expected to increase by up to 70% in case numbers and by 84% in mortality among men by 2050 [21]. In Australia, the state-level cancer registries collate data around individual states and territories, but detailed analyses of this data are minimal. In our current study, which is Australia’s largest on HNC, a detailed analysis of the Queensland Cancer Register data from 1982 to 2018 identified 23,853 adult HNC patients and were mapped for incidence and mortality using the Bayesian approach.

The Bayesian statistical approach is a reliable and established methodology used to map disease incidence and mortality risk within well-defined geographic regions employing LGAs or suburbs as units [14,16]. This retrospective study was the first in Australia to map the head and neck cancer hotspots in Queensland state, Australia, using the Bayesian disease mapping approach. Our study showed that at least 22 LGAs had a significantly higher risk of HNC occurrence, with 12 LGAs having incidence risks above 100% of the Queensland average. Most of the LGAs affected are rural or remote communities.

Demographic factors such as gender are known to influence HNC incidence. Previously, the Northern Territory has reported that approximately five times higher in males compared to females [22]. In our study, HNC incidence was strongly influenced by gender, with median smoothed SIRs for females and males ranging from 0.73 to 1.87 and from 0.54 to 2.80, respectively (Figure 3B,C). A recent retrospective study using the Queensland Oncology Repository (QOR), managed by Cancer Alliance Queensland, focused on data from 2013 to 2015 and reported that 77.7% of HNC patients were men, with 45% being over 65 years of age [23]. These findings align with our study, which found that 77.5% were men, and 43.7% were over 65 years of age. Additionally, marital status has been reported to have a protective effect against metastatic HNC, with married people being less likely to present with metastasis and more likely to receive treatment leading to better survival rates [24]. Similar trends of better outcomes for married people were identified in other studies on cancer in general, and specifically on oral cancer [25,26,27].

Head and neck cancers have previously been reported to have a significant association with treatment-related morbidity and mortality [9]. Our recent study on cancer dataset, focusing on oral cancers (a major subset of HNC), showed that mortality rates have increased in recent years, which can be attributed to factors such as delayed diagnosis and reduced access to services in regional areas of Australia [7]. Additional factors contributing to poorer outcomes after HNC treatment include tumor characteristics and the type and intensity of radiotherapy received, as well as a history of heavy alcohol consumption and limited access due to residence in rural and remote areas [9,23,28,29]. Furthermore, rural residents were reported to travel for treatment and to seek readmission at a different facility from the one that provided the initial treatment [23]. This may be due to the need for frequent travel from rural and remote areas resulting in economic, emotional, and relationship strains [9]. Rurality was also linked to non-treatment for diagnosed HNC in this study, with metropolitan people (9.1%) being less likely to go untreated compared to their rural counterparts (13.7%). This may partly explain the lower overall 2-year survival rates noted in rural cases compared to metropolitan cases (88.7% vs. 75.4%) [23]. Another study involving the Victorian cancer registry explored the role of area-level socio-economic disadvantage on 29 different types of cancers and concluded that 21 types had lower survival rates in more disadvantaged areas [30]. In the case of HNC, inequalities persisted and worsened over the entire period studied (2001–2015), in contrast to many other types of cancers, which showed narrowing disparities [30].

Early detection, prompt referral, and optimal treatment of HNC patients are critical for the commencement of appropriate therapy. In the Australian context, general practitioners (GPs) are invariably the first point of contact for the population and play a pivotal role in fulfilling various functions throughout the cancer care continuum, including facilitating screening and diagnosis, referrals, overseeing the coordination of care delivery, managing the adverse effects of cancer treatment, ensuring survivorship support, and providing palliative care [30,31,32]. In a recent study exploring the geographic variations in referral practices for patients with suspected HNC in two primary healthcare networks in New South Wales, it was reported that the availability of services was a major factor influencing referral decisions among regional GPs, in contrast to patient symptoms being the key factor for metro GPs [32]. Additionally, less than half of the GPs felt that specialists could examine suspected HNC cases within 2 weeks of referral in regional areas, compared to 70% in metro areas [32]. Similar barriers may be expected in regional QLD, which could, in part, explain higher mortality rates in regional areas as reported in our study.

Holistic management of HNC patients typically involves a multidisciplinary team (MDT) of health professionals delivering clinical care focused on therapeutic and supportive interventions [33]. MDTs are known to significantly improve patient adherence and compliance with therapies by preventing or reducing side effects, thereby contributing to better clinical outcomes [34]. However, access to MDTs for rural and regional communities is often challenging and limited due to ongoing health workforce recruitment and retention issues in most countries, including Australia [35,36,37,38]. In this context, the role of cancer or health navigators, particularly in disadvantaged communities, can be a vital link between cancer patients and medical providers in rural and remote communities [39,40,41,42]. In some countries, this role is well established and often performed by a nurse, while in others, non-medical professionals provide support to cancer patients [43,44,45]. In Australia, a critical need for cancer navigators embedded within Aboriginal communities (often residing in rural and remote locations) has been highlighted in multiple studies [46,47,48]. With the new Australian Cancer Nursing and Navigation Program announced recently, expanding the scope of culturally safe programs is warranted [49]. Particularly, the inclusion of activities around improving cancer awareness within these communities and the implementation of navigator-led preventive programs within the Aboriginal communities can contribute to reducing health inequities.

This is the first study in Australia using the Bayesian approach to identify head and neck cancer hotspots. The dataset utilized in this study was obtained from the QCR, a comprehensive register maintained by Cancer Alliance Queensland. This is the largest population-based cancer registry in Australia and is a unique resource that receives mandatory notifications from all public and private hospitals, nursing homes, and pathology laboratories. Hence, the analyzed data represent the most comprehensive dataset that provides an accurate record of cancer in Queensland since its inception in 1982. In contrast, the interactive Australian Cancer Atlas (v2.0) is accessible online and covers a subset of the data across all cancers reported in Australia, including data from 2010 to 2019 for head and neck cancers’ survival rates and diagnosis (https://atlas.cancer.org.au/, accessed on 15 February 2025). However, there are some limitations noted within this retrospective study. The dataset is limited to Queensland state and covers the period from 1 January 1982 to 31 December 2018, making it challenging to generalize trends noted in QLD for the rest of Australia’s states and territories. Also, the diagnosis of HNC was based purely on cases with recorded histological evidence of the type and location of cancer. Additionally, the dataset did not record lifestyle behaviors such as smoking and alcohol consumption history among patients diagnosed with any type of cancer. The lack of this data may limit the conclusions drawn in this study since lifestyle factors have a significant effect on the incidence of multiple head and neck cancers across the region studied. Hence, future studies will need to involve a more comprehensive dataset with potential linkage to different datasets held by multiple custodians, such as the Australian Institute of Health and Welfare, state-level cancer registers, and hospital admissions data.

## 5. Conclusions

This paper confirms the effectiveness of identifying at-risk populations for head and neck cancer within Queensland using the Bayesian disease mapping approach. Furthermore, this modeling has also been able to identify the short-term and overall mortality rates of head and neck cancer within QLD so that targeted prevention programs and interventional strategies can be designed and delivered at the community level identified as at-risk or hotspots, predominately in rural and remote regions. The utilization of cancer navigators to deliver holistic cancer care can be expanded to improve cancer awareness in high-risk regions, thereby enhancing the quality of care and access to services in regional communities.

## Figures and Tables

**Table 1:** Patient cohort characteristics.

Variable	Category	N (%)
Age	<40 years	1349 (5.7)
	40–64 years	12,077 (50.6)
	≥65 years	10,427 (43.7)
Gender	Female	5362 (22.5)
	Male	18,491 (77.5)
Tumor site	Lip	6512 (27.3)
	Oral cavity	6166 (25.8)
	Oropharynx	5118 (21.5)
	Nasopharynx	334 (1.4)
	Hypopharynx	1261 (5.3)
	Larynx	4024 (16.9)
	Nasal cavity and paranasal sinuses	374 (1.6)
	Salivary glands	64 (0.3)
Tumor grade	Well differentiated	4878 (20.5)
	Moderately differentiated	10,985 (46.1)
	Poorly differentiated	4305 (18.0)
	Undifferentiated	84 (0.4)
	Unknown	3601 (15.1)
Status	Alive	9721 (40.8)
	Dead	14,132 (59.2)

## Data Availability

Restrictions apply to the availability of the data. Data were obtained from the Queensland Cancer Register, and forms to request data are available [https://cancerallianceqld.health.qld.gov.au/queensland-cancer-register-qcr/ accessed on 15 February 2025] with the permission of Queensland Health under the PHA 2005.

## Supplementary Materials

The following supporting information can be downloaded at: https://www.mdpi.com/article/10.3390/cancers17050819/s1. LGA level data: Incidence of Head and Neck Cancer (Persons); Incidence of Head and Neck Cancer (Females); Incidence of Head and Neck Cancer (Males); Incidence of Lip cancer; Incidence of Laryngeal cancer; Incidence of Nasopharyngeal cancer; Incidence of Hypopharyngeal cancer; Incidence of Nasal and Paranasal sinus cancer; Incidence of Salivary Gland cancer; Mortality—Head and Neck Cancer (Persons); 3 Year Mortality—Head and Neck Cancer (Persons); 5 Year Mortality—Head and Neck Cancer (Persons).
